# Enrichment of bacteria involved in the nitrogen cycle and plant growth promotion in soil by sclerotia of rice sheath blight fungus

**DOI:** 10.1007/s44154-022-00049-y

**Published:** 2022-08-12

**Authors:** Mirza Abid Mehmood, Yanping Fu, Huizhang Zhao, Jiasen Cheng, Jiatao Xie, Daohong Jiang

**Affiliations:** 1 State Key Laboratory of Agricultural Microbiology, Huazhong Agricultural University, Hubei Province, Wuhan, 430070 China; 2 Plant Pathology, Institute of Plant Protection, MNS University of Agriculture, Multan, 60000 Pakistan; 3 Provincial Key Laboratory of Plant Pathology of Hubei Province, College of Plant Science and Technology, Huazhong Agricultural University, Hubei Province, Wuhan, 430070 China; 4 Hubei Hongshan Laboratory, Wuhan, 430070 China

**Keywords:** Rice sheath blight, *Rhizoctonia solani*, Sclerotia, Reductive nitrogen transformation, Nitrogen fixation, Soil microbiome

## Abstract

Rice sheath blight pathogen, *Rhizoctonia solani*, produces numerous sclerotia to overwinter. As a rich source of nutrients in the soil, sclerotia may lead to the change of soil microbiota. For this purpose, we amended the sclerotia of *R. solani* in soil and analyzed the changes in bacterial microbiota within the soil at different time points. At the phyla level, Proteobacteria, Acidobacteria, Bacteroidetes, Actinobacteria, Chloroflexi and Firmicutes showed varied abundance in the amended soil samples compared to those in the control. An increased abundance of ammonia-oxidizing bacterium (AOB) *Nitrosospira* and Nitrite oxidizing bacteria (NOB) i.e., *Nitrospira* was observed, where the latter is reportedly involved in the nitrifier denitrification. Moreover, *Thiobacillus*, *Gemmatimonas*, *Anaeromyxobacter* and *Geobacter*, the vital players in denitrification, N_2_O reduction and reductive nitrogen transformation, respectively, depicted enhanced abundance in *R. solani* sclerotia-amended samples. Furthermore, asymbiotic nitrogen-fixing bacteria, notably, *Azotobacter* as well as *Microvirga* and *Phenylobacterium* with nitrogen-fixing potential also enriched in the amended samples compared to the control. Plant growth promoting bacteria, such as *Kribbella*, *Chitinophaga* and *Flavisolibacter* also enriched in the sclerotia-amended soil. As per our knowledge, this study is of its kind where pathogenic fungal sclerotia activated microbes with a potential role in N transformation and provided clues about the ecological functions of *R*. *solani* sclerotia on the stimulation of bacterial genera involved in different processes of N-cycle within the soil in the absence of host plants.

**Supplementary Information:**

The online version contains supplementary material available at 10.1007/s44154-022-00049-y.

## Introduction

Rice (*Oryza sativa* L.), the widely consumed staple food, provides 20% of the dietary protein to the growing population in developing countries (FAO, 2004; Pareja et al., 2011). China is the largest producer with 142.3 million tons followed by India with 110.4 million tons production (FAO 2017). Sheath blight disease caused by *Rhizoctonia solani* Kühan (teleomorph: *Thanatephorus cucumeris* (Frank) Donk) in AG-1 is a devastating disease of rice all over the globe (Rao et al., 2020). Due to its special disease symptoms, it is also known as rotten foot stalk, snake skin disease, and mosaic foot disease (Zhang et al., 2019; Molla et al., 2020). In China, the annual disease area is about 15–20 million hm^2^ (González-Vera et al., 2010; Shu et al., 2019). It results in 10–30% yield losses and even up to 50% losses in South China and along the Yangtze River during epidemic years (Yu et al., 2019; Zhu et al., 2019). It is considered as a constant threat to rice-growing areas of South East Asian countries (Cu et al 1996; Shrestha et al 2008; Taheri and Tarighi 2011). Application of nitrogenous fertilizers at high doses and planting of high yield semi-dwarf cultivars resulted in the increased incidence of sheath blight in the past decades (Yellareddygari et al., 2014).

*R. solani* survives in form of sclerotia which are developed by interweaving mycelia on the infected plant and dropped into water or soil (Willetts and Bullock, 1992; Shu et al., 2019; Sun et al., 2020). There is a large number of sclerotia in the soil. A range of 226–636 buoyant and 73–372 non-buoyant *R. solani* sclerotia with varied percentage of viability were detected in crop debris of one-liter soil sample collected from 0–7.6 cm soil depth of severely infected rice fields of Arkansas (Lee, 1980). Sclerotia have strong resistance to stresses and remain viable for 10 months immersed in the paddy soil, soaked in sterile water or placed inside a desiccator (Feng et al., 2017).

Rice-rapeseed rotation is a widely adapted cultivation system in South China. After harvest, sclerotia produced by *R. solani* on rice remain in the soil. For planting rapeseed, the water content of the soil decreased to 60–70% making it suitable for soil microorganisms. Whereas, the aforementioned soil water contents may be challenging for the survival of *R. solani*. We presumed that as a rich source of nutrients, sclerotia may be attacked by soil microorganisms and conversely lead to the change of soil microbiota. To investigate the possible changes of soil microbiota caused by the sclerotia of *R. solani *and screen beneficial microbes, we collected soil from paddy fields, amended sclerotia of *R. solani* and analyzed the changes of bacterial microbiota in the soil at different time points.

## Results

### Preprocessing statistics of 16S sequences

Approximately 7,626,116 raw paired-end and 7,594,659 clean paired-end reads were generated by sequencing platform, and ultimately 6,883,011 sequences were obtained before further processing. Briefly, the quality control statistics showed that the number of reads provided by the platform varied considerably in different treatments during the 3-month study. The clean reads were in the range 252,769–529,423, 262,330–542,472, and 204,315–601,156 during the 1^st^, 2^nd^, and 3^rd^-month soil samples, respectively. The effective read percentage was > 99%, with Q30 values > 83% in all samples (Table S1).

### Rarefaction curve and diversity indices

**Fig. 1:**
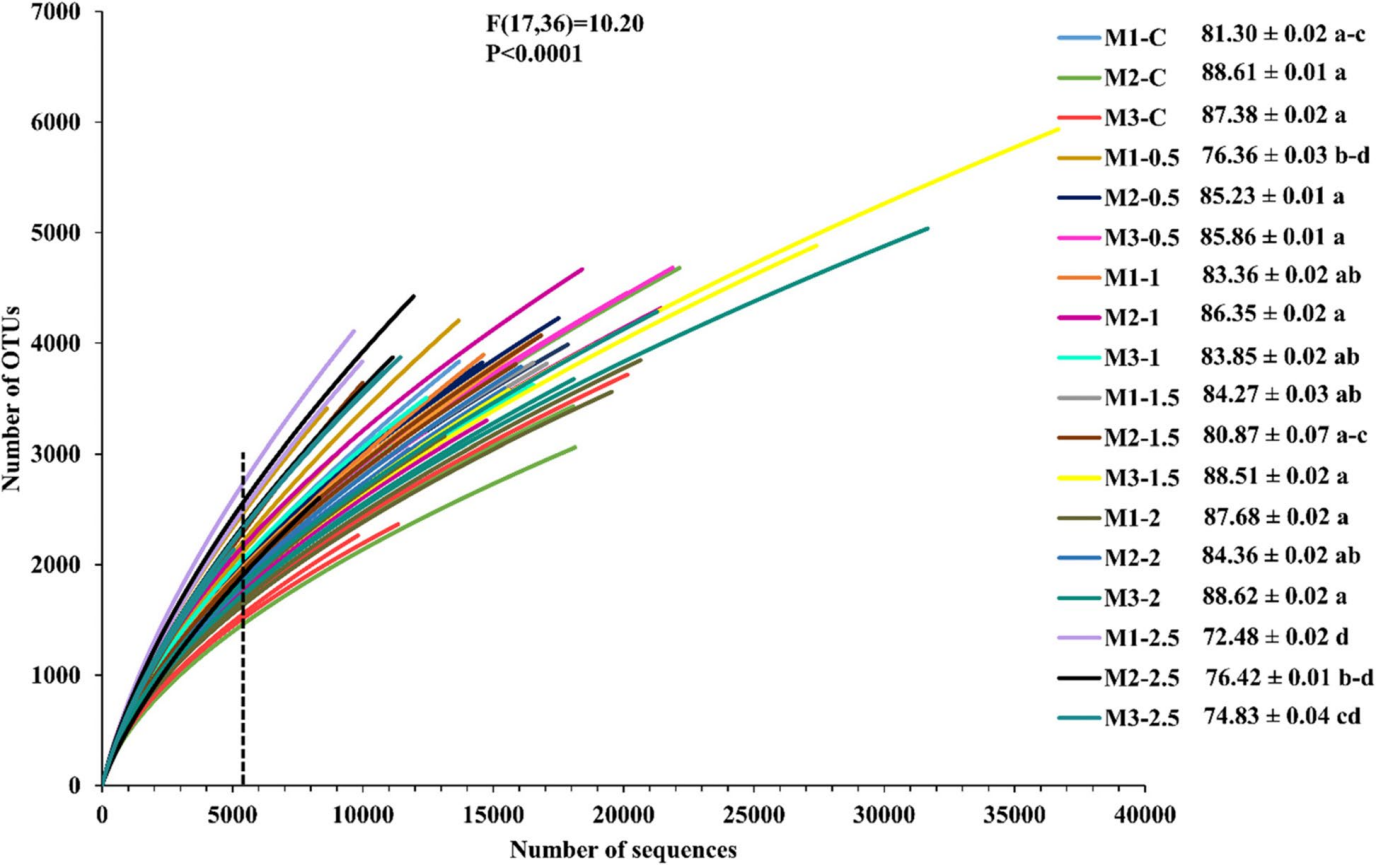
Rarefaction curve of bacterial communities and Good’s coverage estimates (% ± standard deviation) in the non-amended and sclerotia-amended soil samples of rice-rapeseed rotation field soil amended with different doses of *R. solani *sclerotia. M1, M2, and M3 represent 1^st^ month, 2^nd^ month, and 3^rd^ month while C shows the non-amended samples. Moreover, 0.5, 1, 1.5, 2 and 2.5 denotes the different dose of sclerotia in gram amended in soil. The dotted line represents the number of sequences used as a subsample (5400 sequences) for alpha diversity estimates. ANOVA followed by Tukey–Kramer post hoc comparisons was performed using R. Effect of amended and non-amended soil (F(dfn, dfd) and P-value) represented at the top of the graphs while lowercase letters indicate significant differences (P < 0.05)

**Fig. 2:**
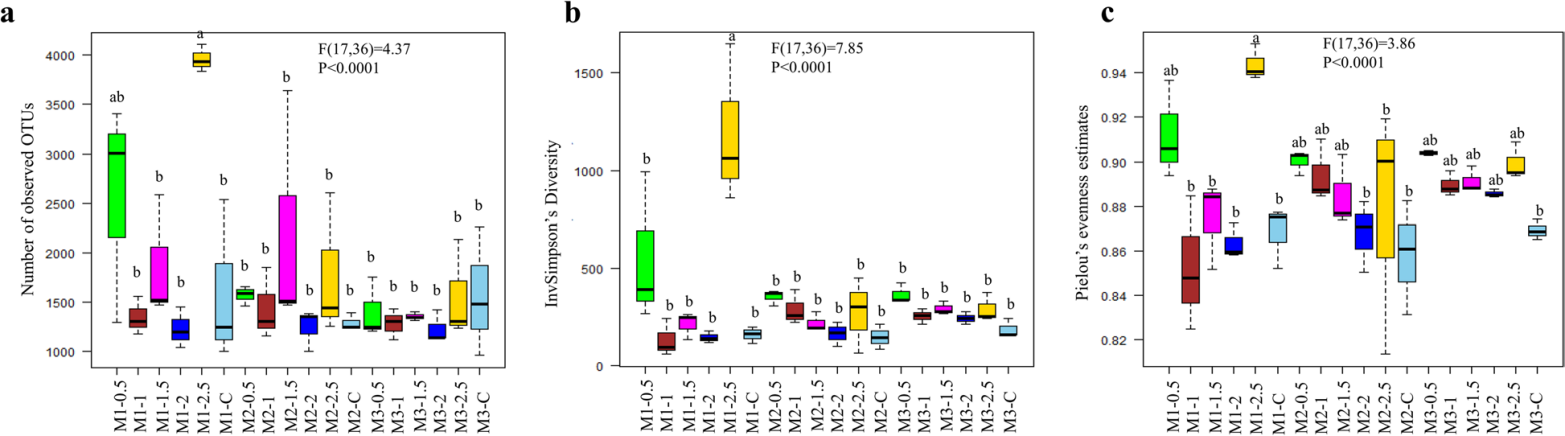
Alpha diversity indices calculated for bacterial communities between the non-amended and sclerotia-amended soil samples in rice-rapeseed rotation field soil amended with different doses of *R. solani *sclerotia. M1, M2, and M3 represent 1^st^ month, 2^nd^ month, and 3^rd^ month while C shows non-amended samples. Moreover, 0.5, 1, 1.5, 2 and 2.5 denotes the different dose of sclerotia in gram amended in soil. ANOVA followed by Tukey–Kramer post hoc comparisons was performed using R. Effect of the amended and non-amended soil (F(dfn, dfd) and *P*-value) represented at the top of the graphs while lowercase letters indicate significant differences (*P* < 0.05)

Rarefaction curves about total bacterial sequences and total OTUs (≥ 97% similarity) in each sample were constructed. Good’s coverage scores were also represented with a rarefaction curve based on 10,000 iterations using mothur. Non-amended samples’ coverage ranging from 81.30–88.61% and 72.48–88.62% was attained in 16S rRNA sequencing of sclerotia-amended soil samples (Fig. 1). In the case of observed OTUs, non-amended samples showed saturation around 950–2550 OTUs while sclerotia-amended samples depicted saturation around 1000–4100. Moreover, the maximum number of observed OTUs was observed in M1-2.5 where the saturation was around 3800–4100 (Fig. 2 a). According to the observed OTUs, InvSimpson’s diversity and evenness, there were no significant differences neither among the amended samples and the control nor among the different time points, except in sample M1-2.5 (Fig. 2b and c).

**Fig. 3:**
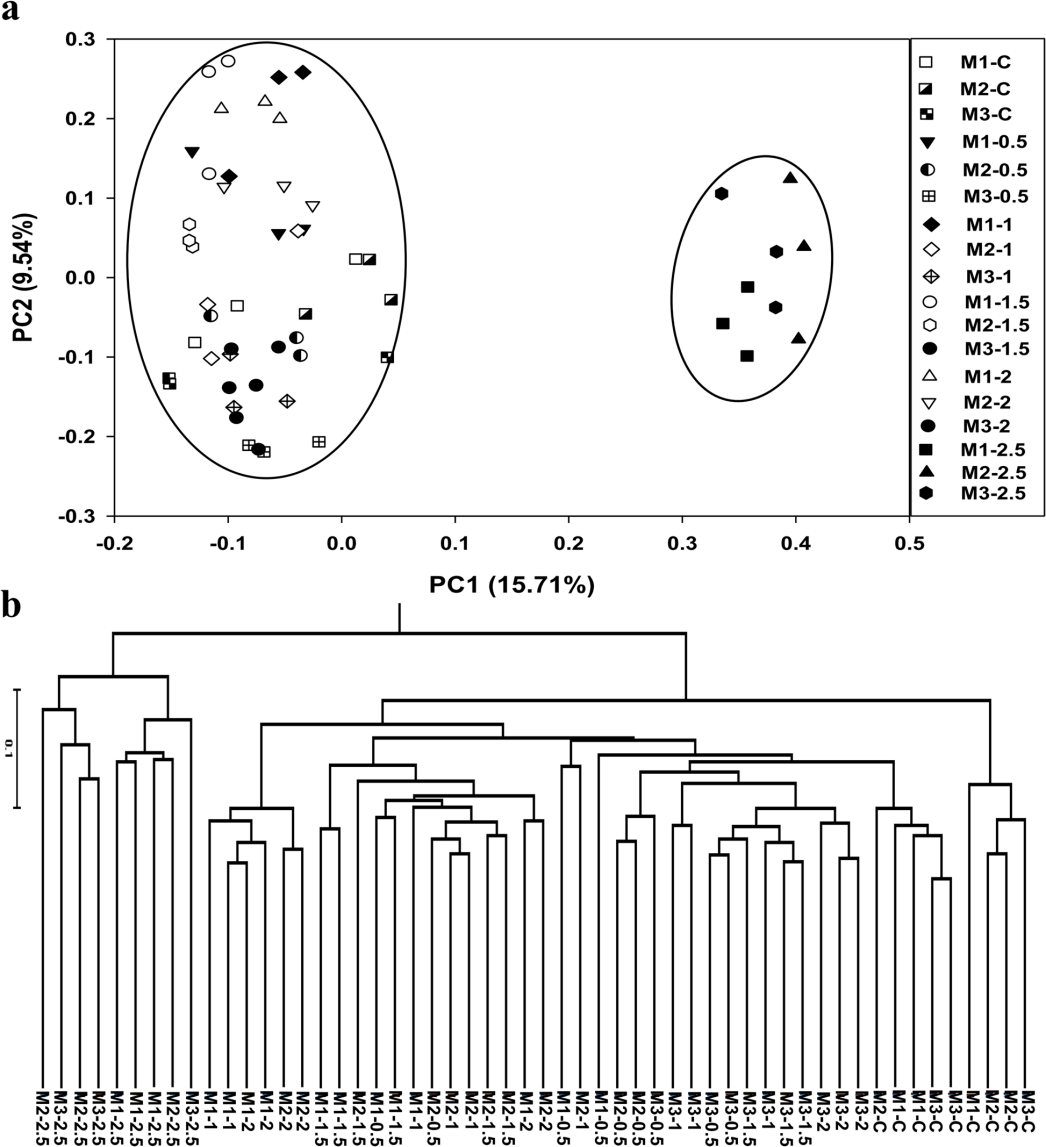
Principal coordinate analysis (PCoA) and hierarchical clustering of bacterial communities in the non-amended and sclerotia-amended soil samples within rice-rapeseed rotation field soil amended with different doses of *R. solani* sclerotia. M1, M2, and M3 represent 1^st^ month, 2^nd^ month, and 3^rd^ month while C shows the non-amended samples. Moreover, 0.5, 1, 1.5, 2 and 2.5 denotes the different dose of sclerotia in gram amended in soil

PCoA of bacterial communities showed 2 clear clusters where all the non-amended and sclerotia-amended samples except the soil samples amended with 2.5 g sclerotia were clustered together and the latter formed a separate cluster. Moreover, both non-amended and amended samples were sparser during the 3-month study. Notably, PC1 showed 15.71% of total variation while PC2 presented 9.54% variation (Fig. 3 a). Hierarchical clustering depicted the same 2 clear clusters where soil samples amended with 2.5 g dose clustered separately as observed in PCoA (Fig. 3 b). Permanova of bacterial communities exhibited statistically significant results among the amended and non-amended samples. Similarly, microbial populations between three months and interaction of the non-amended samples with amended ones for three months also revealed a significant effect in the case of 16S sequencing (Table 1). Subsequently, a multilevel comparison of Months showed that bacterial communities in M1 vs M3 and M2 vs M3 were statistically significantly different. Furthermore, a comparison of different doses with 2.5 g and control also exhibited a statistically significant difference in population except 0.5 vs C (Table S2).

**Table 1:** Permanova of bacterial communities based on Bray–Curtis dissimilarities between non-amended and sclerotia-amended soil samples of rice-rapeseed rotation field soil amended with different doses of *R. solani* sclerotia

Effects	Df	Sum of Squares	Mean Squares	F-value	R^2^	*P*-value
Months	2	0.674	0.337	3.131	0.082	< 0.0001
Doses	5	2.254	0.451	4.191	0.274	< 0.0001
Months × Dosess	10	1.417	0.142	1.317	0.172	< 0.001
Residuals	36	3.872	0.108			

Doses = C (non-amended), 0.5, 1.0, 1.5, 2.0 and 2.5 g sclerotia-amended soil samples

### Relative abundance (%) of different bacterial phyla

Bacterial communities classified to various phyla at ≥ 97% similarity showed varying degrees of increase or decrease in relative abundance (%) of the non-amended and amended samples. Overall, bacterial communities were divided into core bacterial phyla and others (with minor relative abundance). Microbes that did not classify to any phyla were presented in bacteria unclassified. Of the 24 bacterial phyla, 8 core phyla contributed > 92% in total relative abundance.

**Fig. 4:**
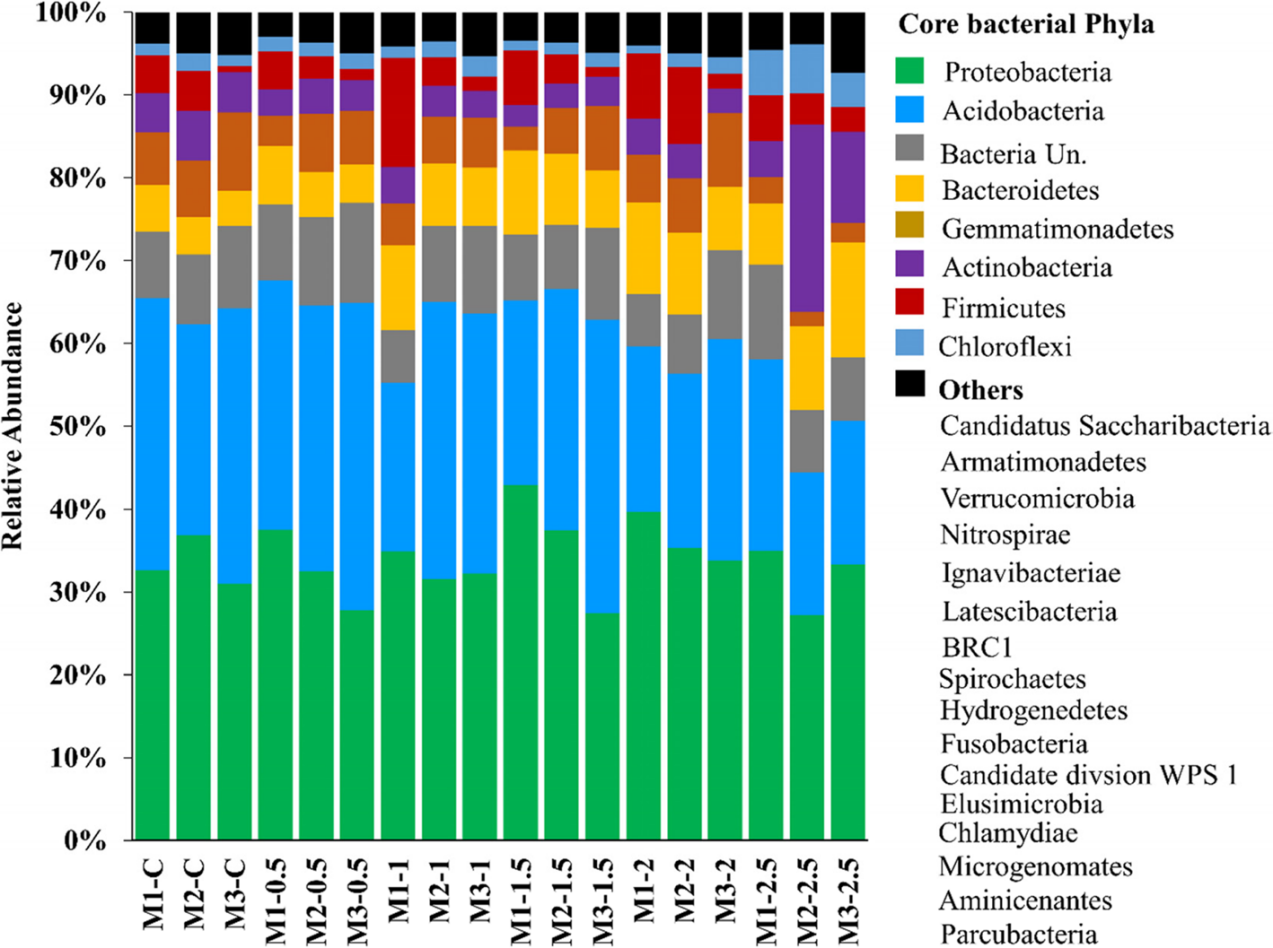
Relative abundance (%) of bacterial phyla depicting increase or decrease in the non-amended and sclerotia-amended soil samples in rice-rapeseed rotation field soil amended with different doses of *R. solani *sclerotia detected using 16S rRNA sequencing. M1, M2, and M3 represent 1^st^ month, 2^nd^ month, and 3^rd^ month while C shows the non-amended samples. Moreover, 0.5, 1, 1.5, 2 and 2.5 denotes the different dose of sclerotia in gram amended in soil

Proteobcteria and Acidobacteria revealed maximum relative abundances individually and together in amended and non-amended samples. The relationship of Acidobacteria abundance and dose of amended sclerotia is inversely proportional with one exception. Amendment of sclerotia significantly stimulated the population of Bacteroidetes and results in 1.3–3.3-fold increase in the third month of 2.5 g sclerotia amended samples compared to the control. The population of Gemmatimonadetes in the soil kept stable while 2.5 g sclerotia amended samples significantly reduced the accumulation of Gemmatimonadetes. The accumulation of Actinobacteria in all sclerotia-amended soil decreased slightly, with an exception of 2.5 g sclerotia-amended soil in which these bacteria were enriched significantly by 2.8-fold and 1.3-fold in the second and third month, respectively. Furthermore, Firmicutes showed decreased abundance during the second and third month at all samples compared to the first month samples, and the relative abundance at the third month was higher in the experimental groups than in the control group. Whereas, the maximum accumulation of Chloroflexi was evident in 2.5 g sclerotia-amended soil where we found 3.79, 2.77, and 3.03-fold increase in abundance compared to the control (Fig. 4).

### Impact of presence or absence of fungal sclerotia on bacterial diversity at the genus level

**Fig. 5:**
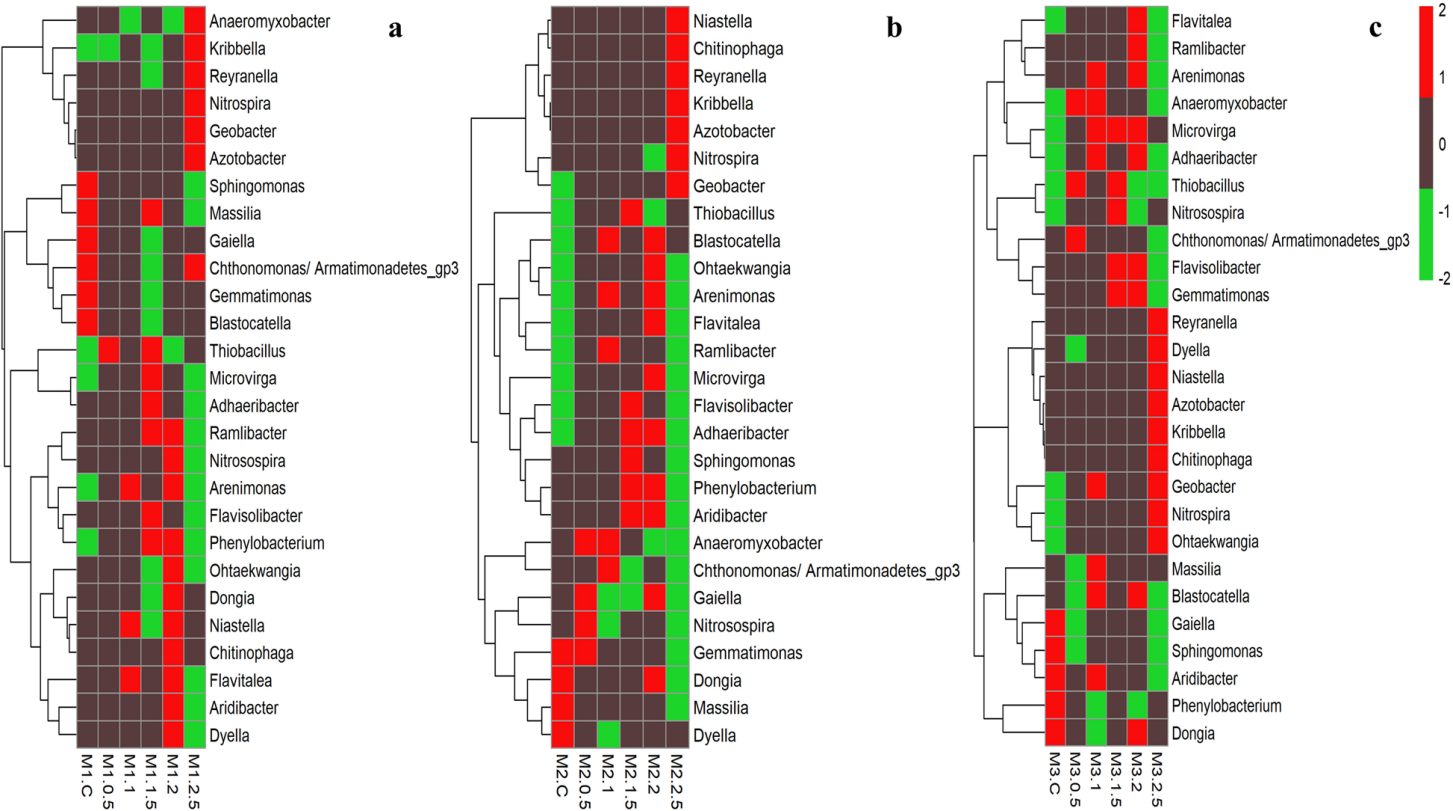
Differentiation of most abundant core bacterial genera illustrating a significant shift in abundance within the non-amended and *R. solani* sclerotia amended soil samples during 1^st^ (**a**), 2^nd^ (**b**) and 3^rd^ (**c**) month as represented by Euclidean distance within rice-rapeseed rotation field soil. M1, M2, and M3 represent 1^st^ month, 2^nd^ month, and 3^rd^ month while C shows the non-amended samples. Moreover, 0.5, 1, 1.5, 2 and 2.5 denotes the different dose of sclerotia in gram amended in soil

After observing a marked shift in the bacterial community at the phyla level, we checked the impact of different doses of* R. solani* sclerotial amendment at the genus level (≥ 97% similarity). Of the top 400 OTUs, 27 representative bacterial genera including both N-cycling bacteria and plant growth promoting bacteria were obtained, ultimately subjected to cluster analysis based on their relative abundance % as represented by Euclidean distance (Fig. 5 and Table S3).

The brief description about enrichment or decline in the abundance of bacterial genera involved in N-cycling (such as *Azotobacter, Gemmatimonas, Anaeromyxobacter, Nitrosospira, Nitrospira, Phenylobacterium* and *Thiobacillus*) and plant growth promoting bacteria (such as *Kribbella, Chitinophaga* and *Flavisolibacter*) in sclerotia-mixed soil and control soil is given below.

Sclerotia amendment declined the accumulation of *Gemmatimonas* during the first month followed by the increased abundance in control and amended sample (M2-0.5) and enhanced abundance in amended samples (M3-1.5 & M3-2) only during third month. *Phenylobacterium* was abundant in sclerotia-amended soil samples (M1-1.5, M1-2, M2-1.5, and M2-2) during 1^st^ and 2^nd^ month while control depicted abundance during 3^rd^ month. *Anaeromyxobacter* and *Nitrosospira* enriched significantly in low-dose sclerotia-amended samples compared to the control. The maximum accumulation of *Microvirga* during the first month was detected in all sclerotia-amended samples except 2.5 g sclerotia-amended samples and control. Sclerotia-amended samples revealed an increased abundance of *Thiobacillus, Geobacter, Kribbella, Nitrospira* and *Azotobacter* compared to the control in which these bacteria could not be examined or observed in lower abundance while the maximum accumulation of the latter was observed in 2.5 g sclerotia-amended soil samples. Moreover, *Chitinophaga* exhibited enhanced abundance in 2 g sclerotia-amended soil in 1^st^ month and in 2.5 g sclerotia-amended soil in 2^nd^ and 3^rd^ month while *Flavisolibacter* was enriched in 1.5 g sclerotia-amended soil of 1^st^ and 2^nd^ month samples while 3^rd^ month samples revealed enrichment in 1.5 and 2 g sclerotia-amended soil.

### Indicator species

To discover the bacterial population responsible for differentiation in the non-amended and sclerotia-amended samples, we used indicator species analysis on full community matrix using Indval function for calculation of associations in R Programming language. OTUs classified at the genus level (≥ 97% similarity) based on their relative abundance % in respective samples are given in Table 2. Of the 18 indicator species from the top 400 OTUs, 4 were shared among the non-amended and sclerotia-amended soil samples while 14 OTUs were from different doses of sclerotia-amended soil. It is obvious that *Azotobacter, Nitrospira, Microvirga, Anaeromyxobacter, Gemmatimonas, Thiobacillus*, etc., served as indicator species of different doses of amended samples. While *Massilia, Flavisolibacter, Chthonomonas*/Armatimonadetes_gp3 and *Phenylobacterium* were the indicator species of both amended and non-amended soil samples (Table 2).

**Table 2:** Bacterial indicator species detected in non-amended and soil samples amended with different concentrations of *R. solani* sclerotia within rice-rapeseed rotation field soil

Sr. No	Genus or higher	Indicator value	P-value	C	0.50	1.00	1.50	2.00	2.50	Sample
1	*Kribbella*	0.990	0.0001 ^***^	0.00	0.00	0.00	0.00	0.02	0.82	2.5
2	*Azotobacter*	0.891	0.0001 ^***^	0.00	0.01	0.02	0.01	0.01	0.40	2.5
3	*Niastella*	0.864	0.0001 ^***^	0.00	0.00	0.09	0.02	0.21	0.40	2 + 2.5
4	*Chitinophaga*	0.756	0.0177 ^*^	0.01	0.00	0.02	0.01	0.06	0.36	2 + 2.5
5	*Nitrospira*	0.934	0.0001 ^***^	0.01	0.09	0.16	0.12	0.02	0.00	0.5 + 1 + 1.5
6	*Microvirga*	0.979	0.0003 ^***^	0.02	0.13	0.14	0.46	0.23	0.02	0.5 + 1 + 1.5 + 2
7	*Flavitalea*	0.941	0.0035 ^**^	0.04	0.11	0.12	0.08	0.17	0.02	0.5 + 1 + 1.5 + 2
8	*Adhaeribacter*	0.954	0.0001 ^***^	0.03	0.10	0.09	0.23	0.12	0.02	0.5 + 1 + 1.5 + 2
9	*Anaeromyxobacter*	0.793	0.021 ^*^	0.01	0.09	0.05	0.20	0.04	0.01	0.5 + 1 + 1.5 + 2
10	*Arenimonas*	0.952	0.000 ^***^	0.02	0.04	0.09	0.08	0.11	0.00	0.5 + 1 + 1.5 + 2
11	*Gemmatimonas*	0.902	0.000 ^***^	0.02	0.04	0.09	0.07	0.09	0.01	0.5 + 1 + 1.5 + 2
12	*Thiobacillus*	0.935	0.000 ^***^	0.00	0.18	0.10	0.24	0.01	0.03	0.5 + 1 + 1.5 + 2.5
13	*Geobacter*	0.920	0.000 ^***^	0.01	0.04	0.04	0.05	0.02	0.27	0.5 + 1 + 1.5 + 2.5
14	*Massilia*	0.917	0.000 ^***^	0.12	0.02	0.04	0.04	0.05	0.01	C + 1 + 1.5 + 2
15	*Ohtaekwangia*	0.936	0.003 ^**^	0.03	0.11	0.16	0.12	0.19	0.12	0.5 + 1 + 1.5 + 2 + 2.5
16	*Flavisolibacter*	0.998	0.000 ^***^	0.48	0.71	0.94	1.32	1.07	0.02	C + 0.5 + 1 + 1.5 + 2
17	*Chthonomonas*/Armatimonadetes_gp3	0.932	0.001 ^**^	0.14	0.11	0.09	0.09	0.06	0.01	C + 0.5 + 1 + 1.5 + 2
18	*Phenylobacterium*	0.968	0.000 ^***^	0.10	0.13	0.08	0.13	0.05	0.01	C + 0.5 + 1 + 1.5 + 2

Dufrene-Legendre indicator species analysis (Indval) was used for calculation of associations in R. The data shows indicator species from top 400 OTUs representing average relative abundance in non-amended and sclerotia-amended soil samples Significance levels: *P* ≤ 0.01

^*^ *P* ≤ 0.001

^**^ *P* ≤ 0.0001

^***^ C shows non-amended sample while 0.5, 1, 1.5, 2 and 2.5 denote different concentrations of sclerotia in gram amended in soil

### Month-wise distribution of OTUs

**Fig. 6:**
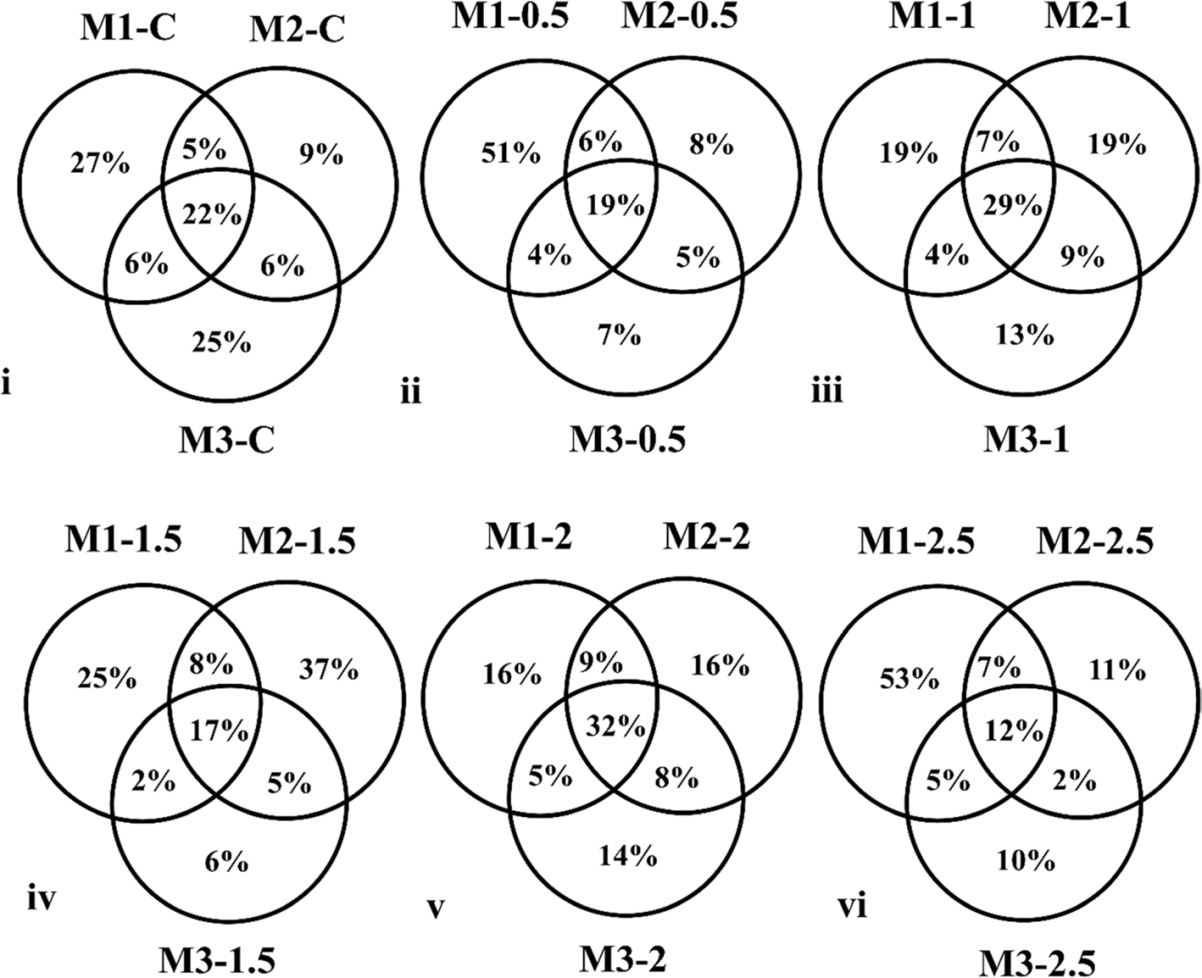
Venn diagram illustrates the percentage of month-wise shared and unique OTUs present in the non-amended and sclerotia-amended soil samples in rice-rapeseed rotation field soil amended with different doses of *R. solani* sclerotia as obtained from 16S rRNA sequencing. M1, M2, and M3 represent 1^st^ month, 2^nd^ month, and 3^rd^ month while C shows the non-amended samples. Moreover, 0.5, 1, 1.5, 2 and 2.5 denotes the different dose of sclerotia in gram amended in soil

The distribution of OTUs among different doses of sclerotia amended soil samples compared with non-amended samples was illustrated using the Venn diagram (Fig. 6). The percentage of unique OTUs increased to 51% and 53% in M1-0.5 and M1-2.5, respectively compared to M1C with 27% unique OTUs. In contrast, all the soil samples incubated with different doses of *R. solani* sclerotia depicted increased unique OTUs distribution during the 2^nd^ month compared with non-amended control except soil samples amended with 0.5 g sclerotia. Moreover, during the 3^rd^ month, all the amended samples exhibited decreased unique OTUs compared with non-amended samples.

## Discussion

Numerous pathogenic fungi produce sclerotia for their survival and *R. solani* is one of the devastating fungi which needs an optimum temperature of 28–32 °C for sclerotial production. *R. solani* sclerotia are produced on rice plants and overwinter in the soil which is rotated with rapeseed along the Yangtze River in China. Sclerotia are a rich source of fiber, protein, chitin, carbohydrate, fat, Ca, K, Mg, and Na (Willettes, 1971; Saito, 1974; Yap et al., 2013; Kong et al., 2016), which can be efficiently utilized by soil-inhabiting microbes by overcoming the fungal defense. In this study, we found that Proteobacteria are known copiotrophic bacteria since they require a carbon-rich environment, thus are considered as indicators of nutrient-rich soil. Firmicutes require a carbon-rich environment, too (Fierer et al., 2007; Lienhard et al., 2014). Acidobacteria is often found in nutrient-poor soil (Smit et al., 2001; Gottel et al., 2011; Beckers et al., 2016). In the present study, compared to the same time point in the non-amended control, Proteobacteria was more abundant while Acidobacteria was less in *R. solani* sclerotia-amended soil samples. Amendment of sclerotia increased the abundance of Firmicutes during first month, while the abundance decreased month-wisely in all the samples. Members of Acidobacteria generally exhibited increased abundance in 3^rd^-month samples compared with the preceding months. Previously, it has been elaborated that Acidobacteria is slow-growing oligotroph (Fierer et al., 2007), which provided us with evidence that increased abundance during the 3^rd^ month might be due to their slow-growing nature. This finding is consistent with the previous study that the members of Actinobacteria can decompose organic matter and improve the agricultural soil (Strap, 2011).

Microbes, plants, and animals require nitrogen which is provided by the nitrogen (N) cycle (Thamdrup, 2012). Almost all eukaryotes and prokaryotes (not including nitrogen-fixing bacteria and archaea) need a fixed form of N e.g. nitrate, ammonium, and monomer dissolved N (amino sugars and amino acids) for their proliferation. Previous studies regarding the physiology of the dissimilatory oxidative and reductive reactions playing role in N-cycling provided evidence of strains chiefly limited to N-fixing bacteria, notably the genera *Bradyrhizobium* and *Azotobacter*, nitrite-oxidizing bacteria (NOB, e.g. the genera *Nitrospira* and *Nitrobacter*), ammonia-oxidizing bacteria (AOB, e.g. *Nitrosospira* and *Nitrosomonas*), and the genera *Azospirillum* and Pseudomonas referred to as heterotrophic denitrifying bacteria (Kaur et al., 2008; Ishii et al., 2011; Inaba et al., 2012; Kondo et al., 2012; Okubo et al., 2012; Fujitani et al., 2013; Ushiki et al., 2013). We perceive that sclerotia being a source of nitrogen after hydrolysis by soil-inhabiting microbes may release different compounds and ammonia which might be due to the activity of AOB and NOB such as *Nitrosospira* and *Nitrospira* and be converted into N_2_O. Other studies revealed that in nitrifier denitrification, oxidation of NH_3_ to NO^-2^ was trailed by reduction through *Nitrosospira* via NO to N_2_O (Colliver and Stephenson 2000; Shaw et al 2006). Some species of *Azospirillum, Bradyrhizobium*, and *Nitrosospira* or *Nitrosomonas* can perform the denitrification process (Rösch et al., 2002; Shaw et al., 2006). Emissions of NO and N_2_O in the soil are primarily associated with denitrification (Houlton and Bai 2009). Several denitrifying bacterial genera have been identified to distribute in different environments (Philippot et al., 2007; Chen et al., 2012). Autotrophic bacteria such as *Thiobacillus denitrifcans *and facultative aerobic heterotrophic bacteria such as *Pseudomonas, Bacillus*, and *Paracoccus* carried out the denitrification (Philippot et al., 2007; Demanèche et al., 2009). We observed that the soil depicted the enhanced abundance of many genera including *Azotobacter, Nitrospira*, and *Nitrosospira* after the addition of sclerotia. The enrichment of these genera depicts their role in N-cycling.

Moreover, *Gemmatimonas* was also found in increased abundance within amended samples. Earlier studies exhibited that *Gemmatimona*s carries out N_2_O reduction (Park et al., 2017). In biological nitrogen fixation, symbiotic and free-living diazotrophic microorganisms reduced the atmospheric nitrogen to reactive and biologically available form (Newton, 2000; Dixon and Kahn, 2004; Franche et al., 2009). It can be symbiotic when plant species and nitrogen-fixing microbes develop a mutualistic association (rhizobia) or asymbiotic when this process is carried out by bacterial genera especially *Azotobacter* and *Beijerinckia* (Freitas, 2007). In mutualistic association, microbes essentially require fixed carbon (especially carbohydrates) for carrying out the process of N fixation (da Silveira et al., 2001; Gross et al., 2004). The enrichment of *Azotobacter* in sclerotia-amended samples might be due to the hydrolysis of cellulose, hemicellulose, and other proximate. Previous studies revealed that paddy soils of China, Japan, and Italy contain *Anaeromyxobacter* and *Geobacter* which make them key players in reductive nitrogen transformation (RNT) (Ding et al., 2015; Kim and Liesack, 2015; Masuda et al., 2017). *Metatranscriptomic* study of paddy soil depicted novel functions of *Geobacter* and *Anaeromyxobacter* in the ecological niche, notably, denitrification support, RNT, and production of NH4^+^ through DNRA and N_2_ fixation (Ueki and Lovley, 2010; Masuda et al., 2017). In the present study, we also observed an increase in the abundance of *Anaeromyxobacter* and *Geobacter* in *R. solani* sclerotia-amended soil samples compared with non-amended control. Moreover, we observed that *Microvirga* (Radl et al., 2014) and *Phenylobacterium* (Yang et al., 2017) with a reported role in nitrogen fixation depicted increased abundance in amended samples during the entire study period except for the latter which exhibited increased abundance during the 1^st^ and 2^nd^ month. The enrichment of these microbes in the amended samples might help increase the nitrogen content of the soil and then promote subsequent rapeseed growth.

In our previous study, we found that several bacterial genera, notably, *Achromobacter, Burkholderia, Chitinophaga, Dyella, Kribbella, Sphingomonas, Mesorhizobium*, and *Rhizobium* with a known potential role as biocontrol agents, plant growth promoters, and biological nitrogen fixation depicted increased abundance in *Sclerotinia sclerotiorum*-amended samples (Mehmood et al., 2020). In this study, we observed that* Kribbella* with a reported role as a biocontrol agent (Igarashi et al., 2017), *Chitinophaga* (Esitken et al., 2005; Yin et al., 2013), *Flavisolibacter* (Xiao et al., 2017), and *Dyella* (Anandham et al., 2008; Palaniappan et al., 2010) with the reported role of plant growth promotion depicted enhanced abundance in *R. solani* sclerotia-amended soil samples. Moreover, several genera including *Gemmatimonas, Phenylobacterium, Anaeromyxobacter, Nitrosospira, Microvirga, Thiobacillus, Azotobacter, Geobacter*, and *Nitrospira* with a known role in nitrogen transformation processes exhibited more abundance in *R. solani* sclerotia amended soil samples. This comparison revealed that different sclerotia producing fungi incited different kinds of bacterial communities in different types of soils which might be due to the preference of microbes.

Conclusively, we perceive that the incubation temperature was ideal for sclerotial germination and multiplication. It provided nutrition to other microbes present in the sclerotiosphere. The microbes involved in plant growth promotion generally and microbes with different roles in nitrogen transformation specifically depicted enrichment in sclerotia-amended samples provided evidence that these microbes utilized the nutrients present in the sclerotia. Our study changed the perspective about the pathogenic fungal sclerotia that for a long time considered to play a negative role related to the plant and its ecological niche. The presence of activated bacterial genera with a potential role in nitrogen transformation in rice-rapeseed rotation field soil could improve soil health, ultimately exert a positive impact on rapeseed crops. It helps us explore the reasons for the successful adoption of rice-rapeseed rotation in Southern China. This study will change the perspective of scientists about the possible functions of pathogenic microbes’ especially fungal sclerotia in the soil.

## Materials and methods

### Soil samples preparation and amendment of sclerotia

The soil of a rice-rapeseed rotation field (upper 20 cm depth) in Shayang County, Hubei Province, P.R. China was collected from five different spots in November 2016. The pH, total nitrogen and carbon contents of soil were 6.78, 0.2% and 1.66%, respectively. Equal quantities of the soil samples were thoroughly mixed to get a composite sample. Soil samples were dried at room temperature subsequently, small roots were removed with the help of forceps, and samples were passed through a 2 mm mesh size sieve as explained by others (Steinbeiss et al., 2008; Mehmood et al., 2020).

*R.* *solani* sclerotia were produced using the method described previously (Zhou et al., 2002). Peeled, sliced and diced potatoes were autoclaved in a 500 mL flask at 121 °C for 60 min. Three agar plugs of a pure culture of *R. solani* strain WH-1, the pathogen of rice sheath blight disease, were transferred to the sterilized flasks containing potato cubes, incubated at 28 ± 2 °C for four weeks, and the mature sclerotia were subsequently dried at room temperature.

Different doses of *R.* *solani* sclerotia i.e., 0.50, 1.00, 1.50, 2.00, or 2.50 g were amended in 100 g rice-rapeseed rotation field soil separately and the soil without sclerotia served as the non-amended control. All the non-amended and amended samples were incubated at 28 ± 2 °C with 12 h/12 h day/night photoperiod in a growth chamber for three months. During the entire experimental period, the water-filled pore space (WFPS) of the soil samples were maintained to 60–80% by weighing the pots and adding sterilized distilled water (Lin et al., 2013; Mehmood et al., 2020). Every month, approximately 10 g of soil from each sample was collected and preserved at -80 °C. The non-amended samples of 1^st^, 2^nd^, and 3^rd^ month were designated as M1-C, M2-C, and M3-C, respectively, while soil samples amended with different doses of *R.* *solani* sclerotia were presented by M1, M2, and M3 followed by their doses (0.50, 1.00, 1.50, 2.00, and 2.50 g). All the samples were maintained in three biological replicates for each month.

### Extraction of soil DNA, amplification and sequencing

DNA was extracted from each replicated soil sample using HiPure Soil DNA Mini Kit (Magen, Guangzhou, China) as per the manufacturer’s instructions. Quantification of the extracted DNA concentration was checked using a Qubit 2.0 Fluorometer (Invitrogen, Carlsbad, CA, USA). Moreover, V3 and V4 hypervariable regions of bacterial 16S ribosomal RNA were targeted using forward and reverse primer with sequences “CCTACGGRRBGCASCAGKVRVGAAT” and “GGACTACNVGGGTWTCTAATCC” specially designed by GENEWIZ (GENEWIZ Inc., South Plainfield, NJ, USA) (Caporaso et al., 2011; Yang et al., 2016; Mehmood et al., 2020). Furthermore, to get the uniform amplification of libraries, 16S rRNA primers were supplemented with indexed adapter sequences. The total reaction volume and thermal cycling conditions for PCR were followed as used in our earlier study (Mehmood et al., 2020). Briefly, TransStart Buffer, TransStart Taq DNA polymerase 2.5 U/ µL, 2.5 mM each dNTPs, primer mix, and 20 ng DNA. Moreover, the thermal cycling conditions consisted of 24 denaturation cycles set at 95 °C for 5 s whereas, denaturation, annealing, and elongation were performed at 94 °C for 180 s, 57 °C for 90 s and 72 °C for 10 s, respectively and finally at 73 °C for 5 min. Subsequently, the amplification product was subjected to electrophores. DNA libraries were constructed as elaborated by Zhao et al. (2017), their quality and quantity were checked using an Agilent 2100 Bioanalyzer (Agilent Technologies, Palo Alto, CA, USA) and Qubit 3.0 Fluorometer, respectively. Subsequently, Illumina MiSeq (Illumina, San Diego, CA, USA) platform at GENEWIZ, Inc. (Suzhou, China) was used for loading multiplexed DNA libraries as per the manufacturer’s defined protocol. Sequencing of 16S rRNA was carried out using PE250/300 paired-end, whereas, image analysis and base calling were performed using MiSeq Control Software (MCS).

### Sequence analysis

Sequenced data consisted of primers, adapters, barcodes, and low-quality reads which were culled from raw data using Cutadapt (version 1.9.1) as described previously (Martin, 2011; Bokulich et al., 2012; Mehmood et al., 2020). Subsequently, the data were subjected to analysis using a pipeline within mothur software package (v. 1.39.5) following the standard operating protocol as given on mothur website (http://www.mothur.org/wiki/MiSeq_SOP) (Schloss et al., 2009). Initially, the make.contigs command was used to join the clean forward and reverse reads keeping in view that any sequence without meaningful overlap between sequences and any contig with an ambiguous base (N) were removed. Moreover, sequences depicting homopolymer > 8 bases were also culled followed by sequences with < 225 bp length with a minimum score of Q30 were further trimmed to obtain trimmed sequences (Jaiswal et al., 2017). Alignment of sequences with reference database i.e. Greengenes (v13_8_99) was performed and the sequences aligned to the incorrect positions were discarded (Schloss, 2009, 2010, 2013). Furthermore, the trimming of sequences was done to make sure that all sequences started and ended at the same alignment coordinates (Schloss, 2013). Later, we identified the unique sequences and their frequency, and these sequences were further denoised within each sample by employing a Single Linkage Preclustering algorithm as used earlier (Huse et al., 2010). Screening of sequences for chimeras was done using the UCHIME algorithm, subsequently, the Gold database was used to compare the obtained sequences (Haas et al., 2011; Edgar et al., 2011).

To taxonomically classify each sequence, we used a naive Bayesian classifier and only high quality and non-chimeric sequences employed against Ribosomal Database Project (RDP) 16S rRNA gene training set (version 10) that includes rRNA gene sequences with a minimum 0.80 threshold value (Wang et al., 2007). The sequences classified as Chloroplasts, Eukaryota, Archaea, and Mitochondria or did not classify to the kingdom level were removed. Moreover, these sequences were used to calculate the pairwise distances followed by the creation of distance matrices. Likewise, a majority consensus taxonomy was assigned to the operational taxonomic units (OTUs) with ≥ 97% similarity thresholds using the average neighbor clustering algorithm (Schloss and Westcott, 2011; Kozich et al., 2013). Removal of singletons was done before normalization of samples based on the smallest sample (Kozich et al., 2013). Afterwards, good’s coverage score, rarefaction curve, and alpha diversity indices i.e. observed OTUs, InvSimpson's diversity, and Pielou’s evenness estimates (Pielou, 1966) were calculated based on 5400 sequences obtained after normalization in mothur with 10,000 iterations.

### Statistical analysis

R-programming language version 3.4.1 was used to carry out statistical analysis (Team RDC, 2017). The differential bacterial OTUs relative abundance was identified using the Kruskal–Wallis rank-sum test without P-value adjustment with the help of mothur. Moreover, significant differences in observed OTUs, InvSimpson’s diversity, and evenness estimates within non-amended and soil samples amended with different doses of *R. solani* sclerotia were tested using ANOVA followed by Tukey’s honest significant difference test for post-hoc comparisons. Bray–Curtis dissimilarity matrices were used to perform hierarchical clustering (group average linkage) and principal coordinate analysis (PCoA) within mothur and Sigmaplot version 12.5 (Systat Software, San Jose, CA) was used for illustrations. Furthermore, the permutational analysis of variance (PERMANOVA) was performed with vegan package (version 2.5–1) having adonis and vegdist functions based on Bray–Curtis dissimilarity matrices within R software to find out the variations in bacterial communities within soil samples amended with different doses of *R. solani* sclerotia and non-amended ones during 3 months (Anderson, 2001; Bach et al., 2018). To find out the OTUs linkage with non-amended and soil samples amended with different doses of *R. solani* sclerotia, the bacterial communities were studied using IndicSpecies package with multipatt function (De Ca´ceres M, Legendre P, , 2009). Cluster analysis of bacterial community based on Euclidean distance was performed using R package pheatmap (version 1.0.8) to identify the differences in non-amended and soil samples amended with different doses of *R. solani* sclerotia (Kolde, 2015).

## Supplementary Information

**Additional file 1: Table S1.** Preprocessing statistics of raw, cleanand trimmed reads of non-amended and soil samples amended with differentconcentrations of *R. solani* sclerotia within rice-rapeseed rotation field soil as shown by 16S rRNA sequencing. **Table S2.** Pairwise multilevel comparison of different effects between the non-amended and sclerotia-amended soil samples of rice-rapeseed rotation field soil amended with different doses of *R.solani* sclerotia. **Table S3.** Different bacterial genera depicted increased or decreased relative abundance (%) in non-amended and soil samples amended with different concentrations of *R.solani* sclerotia within rice-rapeseed rotation field soil.

## Data Availability

All data generated or analysed during this study are included in this published article.
