# Supplementary material for: Enrichment of bacteria involved in the nitrogen cycle and plant growth promotion in soil by sclerotia of rice sheath blight fungus
Source: Stress Biol. 2022 Aug 12;2(1):32. doi: 10.1007/s44154-022-00049-y (PMC10441917; doi:10.1007/s44154-022-00049-y)
Supplement: Supplementary file 1 — Additional file 1: Table S1. Preprocessing statistics of raw, cleanand trimmed reads of non-amended and soil samples amended with differentconcentrations of R. solani sclerotia within rice-rapeseed rotation field soil as shown by 16S rRNA sequencing. Table S2. Pairwise multilevel comparison of different effects between the non-amended and sclerotia-amended soil samples of rice-rapeseed rotation field soil amended with different doses of R.solani sclerotia. Table S3. Different bacterial genera depicted increased or decreased relative abundance (%) in non-amended and soil samples amended with different concentrations of R.solani sclerotia within rice-rapeseed rotation field soil. [file 44154_2022_49_MOESM1_ESM.docx]

**Table S1 Preprocessing statistics of raw, clean and trimmed reads of non-amended and soil samples amended with different concentrations of *R. solani* sclerotia within rice-rapeseed rotation field soil as shown by 16S rRNA sequencing**

| **Sample** | **Raw reads**  **(No.)** | **Clean reads**  **(No.)** | **Effective reads**  **(%)** | **Trimmed sequences**  **(No.)** | **Q30**  **(%)** | **GC content**  **(%)** |
| --- | --- | --- | --- | --- | --- | --- |
| M1-C | 421724 | 418880 | 99.33 | 353318 | 84.43 | 56.36 |
| M2-C | 544378 | 542472 | 99.65 | 504282 | 91.66 | 56.28 |
| M3-C | 329918 | 328572 | 99.58 | 310425 | 92.52 | 56.59 |
| M1-0.5 | 403022 | 399518 | 99.10 | 330511 | 83.18 | 56.20 |
| M2-0.5 | 469633 | 468175 | 99.69 | 431950 | 90.89 | 56.68 |
| M3-0.5 | 430519 | 429408 | 99.74 | 402904 | 91.95 | 56.90 |
| M1-1 | 532470 | 529423 | 99.45 | 460786 | 86.71 | 55.45 |
| M2-1 | 512692 | 510528 | 99.56 | 472960 | 91.17 | 56.42 |
| M3-1 | 305237 | 304481 | 99.75 | 289525 | 92.87 | 56.39 |
| M1-1.5 | 432465 | 430973 | 99.63 | 400535 | 91.01 | 55.41 |
| M2-1.5 | 440739 | 438931 | 99.58 | 392462 | 88.40 | 55.93 |
| M3-1.5 | 602617 | 601156 | 99.76 | 574955 | 93.65 | 56.70 |
| M1-2 | 505512 | 504077 | 99.72 | 475113 | 92.40 | 55.47 |
| M2-2 | 453235 | 451332 | 99.58 | 400750 | 87.78 | 55.70 |
| M3-2 | 518547 | 517319 | 99.76 | 496550 | 93.90 | 56.29 |
| M1-2.5 | 254071 | 252769 | 99.47 | 207209 | 83.51 | 56.33 |
| M2-2.5 | 263847 | 262330 | 99.42 | 212703 | 83.20 | 56.38 |
| M3-2.5 | 205490 | 204315 | 99.42 | 166073 | 83.52 | 55.59 |

Raw Reads: Raw paired-end reads; Clean Reads: Adaptor and below QC removed reads; Effective Reads: (Clean reads/ Raw reads) × 100; Trimmed Reads ≥ 225 bp); Q30: Sequencing error rate in clean reads is < 0.1% in terms of base calling. GC content: G:C ratio. M1, M2 and M3 represent 1^st^ month, 2^nd^ month, and 3^rd^ month while C shows non-amended sample. Moreover, 0.5, 1, 1.5, 2 and 2.5 denote different concentrations of sclerotia in gram amended in soil.

**Table S2. Pairwise multilevel comparison of different effects between the non-amended and sclerotia-amended soil samples of rice-rapeseed rotation field soil amended with different doses of *R. solani* sclerotia**

**Multilevel comparison of Months**

| **Sr. No.** | **Pairs** | **F-value** | **R^2^** | **P-value** | **Adjusted P-value** |
| --- | --- | --- | --- | --- | --- |
| 1 | **M1 vs M2** | 1.474 | 0.042 | 0.071 | 0. 071 |
| 2 | **M1 vs M3** | 3.578 | 0.095 | 0.000 | 0.000 ** |
| 3 | **M2 vs M3** | 1.788 | 0.050 | 0.025 | 0.038 . |

**Multilevel comparison of Concentrations**

| **Sr. No.** | **Pairs** | **F-value** | **R^2^** | **P-value** | **Adjusted P-value** |
| --- | --- | --- | --- | --- | --- |
| 1 | **0.5 vs 1.0** | 1.502 | 0.086 | 0.060 | 0.070 |
| 2 | **0.5 vs 1.5** | 1.422 | 0.082 | 0.091 | 0.098 |
| 3 | **0.5 vs 2.0** | 3.646 | 0.186 | 0.000 | 0.000 ** |
| 4 | **0.5 vs 2.5** | 5.287 | 0.248 | 0.000 | 0.000 ** |
| 5 | **0.5 vs C** | 1.822 | 0.102 | 0.038 | 0.051 |
| 6 | **1.0 vs 1.5** | 1.131 | 0.066 | 0.254 | 0.254 |
| 7 | **1.0 vs 2.0** | 1.779 | 0.100 | 0.041 | 0.051 |
| 8 | **1.0 vs 2.5** | 5.570 | 0.258 | 0.000 | 0.000 ** |
| 9 | **1.0 vs C** | 2.472 | 0.134 | 0.004 | 0.006 * |
| 10 | **1.5 vs 2.0** | 2.930 | 0.155 | 0.002 | 0.004 * |
| 11 | **1.5 vs 2.5** | 5.634 | 0.260 | 0.000 | 0.000 ** |
| 12 | **1.5 vs C** | 2.901 | 0.153 | 0.000 | 0.000 ** |
| 13 | **2.0 vs 2.5** | 6.696 | 0.295 | 0.000 | 0.000 ** |
| 14 | **2.0 vs C** | 3.589 | 0.183 | 0.000 | 0.000 ** |
| 15 | **2.5 vs C** | 5.247 | 0.247 | 0.000 | 0.001 ** |

Significance levels: 0.0001 ‘***’ 0.001 ‘**’ 0.01 ‘*’ 0.05 ‘.’

**Table S3 Different bacterial genera depicted increased or decreased relative abundance (%) in non-amended and soil samples amended with different concentrations of *R. solani* sclerotia within rice-rapeseed rotation field soil**

| **Sr. No.** | **Genus or higher** | ****P*-value** | **M1-C** | **M1-0.5** | **M1-1** | **M1-1.5** | **M1-2** | **M1-2.5** | **M2-C** | **M2-0.5** | **M2-1** | **M2-1.5** | **M2-2** | **M2-2.5** | **M3-C** | **M3-0.5** | **M3-1** | **M3-1.5** | **M3-2** | **M3-2.5** |
| --- | --- | --- | --- | --- | --- | --- | --- | --- | --- | --- | --- | --- | --- | --- | --- | --- | --- | --- | --- | --- |
| 1 | *Sphingomonas* | 0.004 | 4.04 | 2.22 | 2.88 | 2.89 | 2.51 | 0.45 | 2.04 | 1.44 | 2.12 | 4.34 | 2.39 | 0.58 | 2.77 | 1.14 | 2.35 | 1.76 | 2.27 | 0.69 |
| 2 | *Flavisolibacter* | 0.000 | 0.64 | 1.01 | 1.31 | 1.76 | 1.35 | 0.01 | 0.37 | 0.63 | 0.81 | 1.40 | 1.01 | 0.03 | 0.42 | 0.48 | 0.71 | 0.79 | 0.85 | 0.00 |
| 3 | *Gemmatimonas* | 0.001 | 0.78 | 0.31 | 0.30 | 0.22 | 0.29 | 0.32 | 0.82 | 0.69 | 0.48 | 0.37 | 0.46 | 0.24 | 0.84 | 0.71 | 0.80 | 0.98 | 1.02 | 0.33 |
| 4 | *Phenylobacterium* | 0.002 | 0.22 | 0.47 | 0.50 | 0.68 | 0.70 | 0.14 | 0.31 | 0.25 | 0.31 | 0.36 | 0.37 | 0.09 | 0.25 | 0.17 | 0.11 | 0.20 | 0.12 | 0.20 |
| 5 | *Aridibacter* | 0.002 | 0.28 | 0.23 | 0.37 | 0.35 | 0.43 | 0.01 | 0.17 | 0.16 | 0.21 | 0.28 | 0.26 | 0.04 | 0.22 | 0.11 | 0.21 | 0.18 | 0.18 | 0.04 |
| 6 | *Blastocatella* | 0.125 | 0.36 | 0.17 | 0.24 | 0.12 | 0.20 | 0.20 | 0.09 | 0.14 | 0.27 | 0.19 | 0.25 | 0.15 | 0.23 | 0.12 | 0.25 | 0.17 | 0.27 | 0.14 |
| 7 | *Anaeromyxobacter* | 0.011 | 0.19 | 0.41 | 0.15 | 0.29 | 0.14 | 0.69 | 0.16 | 0.30 | 0.29 | 0.15 | 0.08 | 0.08 | 0.04 | 0.15 | 0.15 | 0.09 | 0.09 | 0.06 |
| 8 | *Nitrospira* | 0.005 | 0.10 | 0.12 | 0.08 | 0.04 | 0.04 | 0.48 | 0.09 | 0.21 | 0.10 | 0.13 | 0.05 | 0.39 | 0.11 | 0.21 | 0.22 | 0.24 | 0.29 | 0.35 |
| 9 | *Chthonomonas/ Armatimonadetes*_gp3 | 0.014 | 0.16 | 0.05 | 0.04 | 0.03 | 0.05 | 0.11 | 0.13 | 0.13 | 0.16 | 0.08 | 0.10 | 0.08 | 0.29 | 0.33 | 0.22 | 0.31 | 0.31 | 0.09 |
| 10 | *Microvirga* | 0.001 | 0.02 | 0.28 | 0.26 | 1.14 | 0.25 | 0.00 | 0.04 | 0.07 | 0.11 | 0.19 | 0.39 | 0.03 | 0.02 | 0.03 | 0.05 | 0.05 | 0.05 | 0.03 |
| 11 | *Gaiella* | 0.002 | 0.14 | 0.11 | 0.11 | 0.05 | 0.08 | 0.10 | 0.13 | 0.15 | 0.10 | 0.10 | 0.16 | 0.10 | 0.22 | 0.14 | 0.19 | 0.20 | 0.19 | 0.16 |
| 12 | *Ohtaekwangia* | 0.003 | 0.06 | 0.05 | 0.06 | 0.02 | 0.11 | 0.02 | 0.03 | 0.15 | 0.20 | 0.15 | 0.27 | 0.06 | 0.01 | 0.14 | 0.22 | 0.18 | 0.20 | 0.29 |
| 13 | *Dongia* | 0.004 | 0.03 | 0.02 | 0.08 | 0.00 | 0.15 | 0.01 | 0.30 | 0.05 | 0.05 | 0.05 | 0.25 | 0.02 | 0.21 | 0.18 | 0.05 | 0.13 | 0.23 | 0.14 |
| 14 | *Thiobacillus* | 0.002 | 0.00 | 0.18 | 0.06 | 0.12 | 0.00 | 0.05 | 0.00 | 0.10 | 0.06 | 0.22 | 0.00 | 0.01 | 0.00 | 0.25 | 0.17 | 0.39 | 0.02 | 0.03 |
| 15 | *Niastella* | 0.002 | 0.01 | 0.01 | 0.24 | 0.00 | 0.39 | 0.06 | 0.00 | 0.00 | 0.01 | 0.02 | 0.17 | 0.64 | 0.00 | 0.00 | 0.00 | 0.03 | 0.06 | 0.50 |
| 16 | *Massilia* | 0.001 | 0.34 | 0.15 | 0.13 | 0.25 | 0.15 | 0.01 | 0.33 | 0.08 | 0.06 | 0.07 | 0.10 | 0.00 | 0.03 | 0.00 | 0.04 | 0.03 | 0.03 | 0.03 |
| 17 | *Flavitalea* | 0.004 | 0.04 | 0.08 | 0.15 | 0.06 | 0.12 | 0.00 | 0.03 | 0.12 | 0.11 | 0.07 | 0.17 | 0.02 | 0.05 | 0.13 | 0.11 | 0.09 | 0.22 | 0.04 |
| 18 | *Adhaeribacter* | 0.018 | 0.05 | 0.16 | 0.10 | 0.46 | 0.08 | 0.01 | 0.03 | 0.11 | 0.10 | 0.18 | 0.20 | 0.01 | 0.00 | 0.03 | 0.09 | 0.05 | 0.07 | 0.02 |
| 19 | *Kribbella* | 0.002 | 0.00 | 0.00 | 0.01 | 0.00 | 0.02 | 0.04 | 0.00 | 0.00 | 0.00 | 0.01 | 0.02 | 1.86 | 0.00 | 0.00 | 0.00 | 0.00 | 0.01 | 0.56 |
| 20 | *Ramlibacter* | 0.002 | 0.10 | 0.09 | 0.08 | 0.12 | 0.14 | 0.02 | 0.07 | 0.11 | 0.15 | 0.10 | 0.12 | 0.04 | 0.07 | 0.07 | 0.08 | 0.06 | 0.09 | 0.04 |
| 21 | *Reyranella* | 0.014 | 0.04 | 0.03 | 0.05 | 0.02 | 0.05 | 0.14 | 0.06 | 0.04 | 0.06 | 0.05 | 0.05 | 0.17 | 0.06 | 0.10 | 0.06 | 0.07 | 0.12 | 0.27 |
| 22 | *Nitrosospira* | 0.147 | 0.07 | 0.09 | 0.07 | 0.08 | 0.10 | 0.02 | 0.07 | 0.11 | 0.05 | 0.07 | 0.07 | 0.05 | 0.05 | 0.07 | 0.07 | 0.09 | 0.05 | 0.06 |
| 23 | *Arenimonas* | 0.001 | 0.02 | 0.05 | 0.11 | 0.10 | 0.13 | 0.00 | 0.01 | 0.06 | 0.12 | 0.10 | 0.14 | 0.00 | 0.02 | 0.02 | 0.05 | 0.03 | 0.06 | 0.01 |
| 24 | *Geobacter* | 0.036 | 0.01 | 0.03 | 0.03 | 0.03 | 0.02 | 0.49 | 0.00 | 0.04 | 0.02 | 0.05 | 0.01 | 0.22 | 0.00 | 0.05 | 0.08 | 0.06 | 0.03 | 0.09 |
| 25 | *Chitinophaga* | 0.026 | 0.00 | 0.00 | 0.06 | 0.01 | 0.11 | 0.00 | 0.01 | 0.00 | 0.01 | 0.01 | 0.05 | 0.24 | 0.01 | 0.00 | 0.01 | 0.01 | 0.02 | 0.82 |
| 26 | *Dyella* | 0.029 | 0.10 | 0.04 | 0.10 | 0.06 | 0.12 | 0.00 | 0.20 | 0.05 | 0.01 | 0.03 | 0.07 | 0.02 | 0.01 | 0.00 | 0.01 | 0.01 | 0.02 | 0.06 |
| 27 | *Azotobacter* | 0.105 | 0.00 | 0.01 | 0.02 | 0.01 | 0.01 | 0.23 | 0.00 | 0.01 | 0.01 | 0.01 | 0.02 | 0.46 | 0.00 | 0.01 | 0.02 | 0.01 | 0.00 | 0.52 |

* *P*-value indicates the result of Kruskal Wallis test without adjustment which accounted for the average relative abundance of different samples to identify microbes differed significantly in soil samples amended with or without different concentrations of *R. solani* sclerotia. M1, M2 and M3 represent 1^st^ month, 2^nd^ month, and 3^rd^ month while C shows non-amended sample. Moreover, 0.5, 1, 1.5, 2 and 2.5 denote different concentrations of sclerotia in gram amended in soil.
